# Sperm mtDNA Copy Number Is Not Associated With Midpiece Size Among Songbirds

**DOI:** 10.1002/ece3.71055

**Published:** 2025-03-02

**Authors:** Laima Bagdonaitė, Quentin Mauvisseau, Arild Johnsen, Jan T. Lifjeld, Erica H. Leder

**Affiliations:** ^1^ Natural History Museum University of Oslo Oslo Norway; ^2^ Tjärnö Marine Laboratory, Department of Marine Sciences University of Gothenburg Strömstad Sweden; ^3^ Section of Ecology and Evolution, Department of Biology University of Turku Turku Finland

**Keywords:** midpiece size, mitochondrial DNA, Passerides, sperm evolution

## Abstract

Tremendous variation in sperm morphology is observed across the animal kingdom. Within avian taxa, the songbirds (infraorder Passerides) have the largest variation in sperm morphology. Their spermatozoa move by using energy generated in the midpiece, which is formed by multiple mitochondria fusing together during spermatogenesis. However, very little is known regarding the number of mitochondria required to form the songbird midpiece. Based on previous research showing an association of midpiece length and mitochondrial DNA (mtDNA) copy number in the zebra finch *Taeniopygia guttata* , we hypothesize that songbird species with longer sperm midpieces have more copies of mtDNA. We estimated the sperm mtDNA copy number in 19 species from 10 families within Passerides, covering a broad range of midpiece sizes. Mitochondrial and nuclear DNA abundance were determined using droplet digital PCR (ddPCR) and the ratio between mitochondrial and single‐copy nuclear genes was used to estimate mtDNA copy number per spermatozoon. We found that species differ in their average mtDNA copy number, but the variation was small and not significantly related to midpiece length. A possible explanation is that mitochondrial genomes are eliminated in the spermatids during spermatogenesis.

## Graphical abstract

We investigated mtDNA copy number in 19 wild passerine species representing more than a 100‐fold variation in midpiece length. Using highly accurate ddPCR quantification technology, we show that wild songbird spermatozoa have a relatively low interspecific variation of mtDNA content.

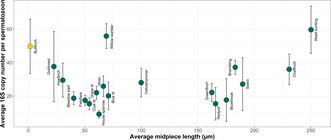

## 1 Introduction

Across the animal kingdom, spermatozoa are known to be among the most diverse cells, despite their similar function—the fertilization of the ova (Pitnick et al. 2009). In songbirds (infraorder Passerides), spermatozoa have a helically coiled head (comprising the acrosome and nucleus), and the midpiece coiled around the flagellum (Jamieson 2007; Koehler 1995). The midpiece houses mitochondria, and in avian sperm, the energy used for the propulsion of the spermatozoa presumably comes mainly from oxidative phosphorylation (OXPHOS) reactions, while glycolic activity seems to be limited (Cummins 2009; Rowe et al. 2013). While the total length of spermatozoa can vary between 40 μm and almost 300 μm across songbirds, the midpiece length can range from as short as 1.7 μm in the Eurasian bullfinch ( *Pyrrhula pyrrhula* ) to nearly 250 μm in the common reed bunting ( *Emberiza schoeniclus* ) (Immler et al. 2011; Omotoriogun et al. 2016) with a general pattern that longer sperm have longer midpieces (Immler and Birkhead 2006). Despite such high variation, its underlying causes are still not fully known, but theory predicts that spermatozoa with increased midpiece size contain more fused mitochondria and therefore produce more energy necessary for the movement of a larger cell (Cardullo and Baltz 1991; Rowe et al. 2013). However, there are alternative hypotheses suggesting that instead of being important for energy production, longer midpieces are essential to provide structural support for longer sperm cells (Lüpold et al. 2009). Every sperm cell has only a single copy of the (haploid) nuclear genome; however, the total number of mitochondria across all birds varies from 20 to ca 2500 per spermatozoon, making the mtDNA copy number also (potentially) highly variable (Jamieson 2007).

The assumption that longer midpieces are formed by more mitochondria remains untested in passerines. In this group, at the late stages of spermatogenesis, mitochondria fuse together and wrap around the flagellum (Aire 2014), therefore making it impossible to quantify the number of mitochondria forming the passerine midpiece using transmission electron microscopy (TEM), commonly used to study sperm ultrastructure. Known exceptions and deviations from the characteristic songbird sperm form with a coiled and elongated midpiece exist in two species of *Pyrrhula* finches (Birkhead et al. 2006; Lifjeld et al. 2013). More recently, a similar case has also been described in the red‐browed finch *Neochmia temporalis temporalis* (Rowe et al. 2024). In all three instances, sperm midpiece is formed by clusters of mitochondria as opposed to one fused mitochondrion (Birkhead et al. 2006; Lifjeld et al. 2013; Rowe et al. 2024). A study on zebra finch spermatozoa with large within‐species variation in midpiece length has shown that longer midpieces have more copies of mtDNA (Knief et al. 2021). The observed difference in mtDNA copy number (expressed as the ratio between mtDNA and autosomal DNA) was rather low: 11.3 in spermatozoa with short midpieces (~25 μm) and 16.9 copies when the midpiece is long (~31 μm) (Knief et al. 2021, 2017). While such results could be linked to an increase in the number of mitochondria that fuse during spermatogenesis, this remains difficult to confirm. Another study on zebra finches investigated the relationship between the length and volume of the midpiece (Mendonca et al. 2018) and found that within the species, longer midpieces became thinner and thus volume remained rather unchanged with length. However, (Cramer et al. 2022) reported that across species with varying midpiece lengths and gyre intervals, the volume and length of the midpiece correlated positively. Thus, we predict that species with longer midpieces, and hence larger midpiece volumes, should have more fused mitochondria and thus a higher mtDNA copy number.

A number of studies in other taxa have investigated mtDNA copy number in spermatozoa and reported that spermatozoa undergo a reduction in copy number, possibly as a mechanism to prevent the transfer of paternal mtDNA to the offspring (Boguenet et al. 2022; DeLuca and O'Farrell 2012; Guo et al. 2017; Luo et al. 2013; Nishimura et al. 2006). In the fruit fly ( *Drosophila melanogaster* ), prezygotic barriers of mtDNA transmission ensure that mature spermatozoa do not possess large amounts of mtDNA (DeLuca and O'Farrell 2012), and similar patterns of mtDNA reduction have been observed in the Japanese medaka fish ( *Oryzias latipes* ) (Nishimura et al. 2006). It is important to note, however, that these studies emphasize that while mtDNA content is reduced, the mitochondria are kept in the sperm cell. Additionally, research on mammals has shown that spermatozoa carrying a higher mtDNA copy number are typically associated with lower quality or speed; therefore, the most motile sperm reduce but do not fully eliminate their mtDNA before fertilization (Boguenet et al. 2022; Guo et al. 2017; Luo et al. 2013). If a similar mtDNA reduction is true in passerines, it may be difficult to infer the initial mitochondrial loading from the copy number of mtDNA in mature spermatozoa.

Here, we aim to explore the relationship between mtDNA copy number and midpiece length across songbird species. The average number of mtDNA copies per spermatozoon was determined using, for the first time, droplet digital PCR (ddPCR) (Bagdonaitė et al. 2024). Representatives from 10 songbird families were selected to ensure a broad range of midpiece morphology.

## 2 Materials and Methods

### 2.1 Samples

A total of 79 unique sperm samples were collected from 19 songbird species in Norway during the breeding seasons of 2021, 2022, and 2023. Birds were caught using mist nets and song playback. A wide range of common breeding species was selected to represent variable sperm midpiece morphology (average midpiece length 1.7–250 μm) (Table 1). Family, common, and scientific species names are provided in Table 1; from here on, in the text and figures, we will use common names, omitting “European,” “Eurasian,” and “Common” (i.e., common reed bunting will be referred to as reed bunting). Information on collection date, as well as the accession numbers for the DNA bank of the Natural History Museum in Oslo, Norway (NHMO), can be found in Table S1.

**TABLE 1:** Species used in this study.

Family	Species	*N*	Average of total sperm length (μm) ± SD	Average of midpiece length (μm) ± SD
Emberizidae	Yellowhammer^a^ *Emberiza citrinella*	4	130.60 ± 2.30	99.93 ± 1.48
	Common reed bunting *Emberiza schoeniclus*	4	279.84 ± 5.37	250.21 ± 5.49
Fringillidae	European greenfinch *Chloris*	4	192.85 ± 5.76	162.44 ± 6.90
	Common redpoll *Acanthis flammea*	4	196.37 ± 4.36	165.77 ± 4.84
	Eurasian siskin *Spinus spinus*	10	218.47 ± 3.88	189.71 ± 4.84
	Eurasian bullfinch *Pyrrhula pyrrhula*	4	50.46 ± 4.75	1.72 ± 0.14
	Hawfinch *Coccothraustes coccothraustes*	4	65.79 ± 2.08	30.57 ± 1.92
	Eurasian chaffinch^a^ *Fringilla coelebs*	4	252.46 ± 4.97	230.88 ± 1.644
	Brambling *Fringilla montifringilla*	4	212.75 ± 4.69	183.12 ± 5.47
Motacillidae	Meadow pipit^a^ *Anthus pratensis*	3	71.14 ± 1.91	40.27 ± 2.57
Muscicapidae	Bluethroat^a^ *Luscinia svecica*	4	209.80 ± 6.05	175.34 ± 3.12
Paridae	Coal tit^a^ *Periparus ater*	4	93.84 ± 2.23	54.00 ± 2.20
	Great tit *Parus major*	4	97.77 ± 2.93	60.40 ± 2.65
	Eurasian blue tit^a^ *Cyanistes caeruleus*	4	105.20 ± 3.20	70.95 ± 1.40
Passeridae	House sparrow^a^ *Passer domesticus*	4	99.39 ± 3.11	62.63 ± 1.13
Phylloscopidae	Willow warbler *Phylloscopus trochilus*	4	93.31 ± 2.01	69.09 ± 1.85
Regulidae	Goldcrest *Regulus regulus*	4	55.32 ± 1.13	22.70 ± 1.00
Troglodytidae	Eurasian wren^a^ *Troglodytes troglodytes*	4	88.13 ± 1.89	66.50 ± 1.62
Turdidae	Fieldfare^a^ *Turdus pilaris*	2	86.04 ± 2.21	50.33 ± 1.42

*Note:* The number of individuals per species used in our analysis is indicated in the table. The average total sperm and midpiece length measurements as well as standard deviation (SD) were obtained from the sperm morphology database of NHMO. *N* is the number of individuals used in the study.

^a^ Average midpiece lengths for these species were calculated from measurements of individuals used in this study.

All samples were collected from males in breeding condition using gentle cloacal massage (Kucera and Heidinger 2018; Wolfson 1952). We followed the same sample cleaning procedure as described in previous works (Bagdonaitė et al. 2024; Kucera and Heidinger 2018). The ejaculate was collected in a microcapillary tube and promptly mixed with phosphate‐buffered saline (PBS) (Kleven et al. 2008). This process was carried out to prevent the sperm cells from clumping and to enable easier pipetting during the DNA extraction phase. After several rounds of mixing the sample with PBS, centrifugation and removal of the supernatant were carried out to ensure that as much debris as possible was removed, after which the samples were frozen at −80°C until DNA extraction. A small aliquot of the cleaned sample was also preserved in formalin and visually inspected for cleanliness, ensuring there was no visible debris or other cells. All sampling was conducted following the ethical guidelines for use of animals in research and with permissions from the local authorities: Norwegian Food Safety Authority (permit no. 23294 and 29575) and The Norwegian Environment Agency (permit no. 2021/39021).

DNA from sperm samples was extracted using the QIAmp DNA Micro Kit (QIAGEN Inc., Valencia, CA, USA), following the protocol described by Kucera and Heidinger (2018) with the changes outlined by Bagdonaitė et al. (2024). In short, frozen sperm samples were mixed with dithiothreitol (DTT) buffer and incubated for 2 h at 65°C to ensure the complete lysis of sperm cell walls and the release of DNA. This was followed by the steps described by Kucera and Heidinger (2018) and the final elution step was done with 25 μL MilliQ filtered water, passing the supernatant through the membrane twice to ensure maximum DNA yield. Once DNA was extracted, total DNA concentration was measured using a Qubit 2.0 fluorometer with the DNA High Sensitivity Assay (Invitrogen). All samples were then diluted to 0.5 ng/μL using MilliQ water before further downstream analysis.

### 2.2 Sperm Morphology

Individual sperm characteristics (total length and midpiece length) were measured from the ejaculate aliquot preserved in formalin, and the species averages were calculated from these measurements. On average, 10 cells per individual were measured to obtain a reliable measurement (Laskemoen et al. 2007). Individual midpiece measurements were available for 48 individuals in 10 of the 19 species. For the remaining samples, we retrieved species averages from the NHMO sperm morphology database (Lifjeld 2019).

### 2.3 Primer Design

To assess mtDNA copy number variation across species, we designed primers targeting two mitochondrial and two nuclear fragments (Table 2). Mitochondrial 16S and 12S were used to quantify mtDNA copy number, while single‐copy nuclear GAPDH and ELF1 were used to quantify nuclear DNA copy number and served as the markers of cell number (Atema et al. 2013; Boguenet et al. 2022). The ratio of mtDNA to nDNA copy number was used to provide a reliable estimate of the number of mtDNA copies per sperm cell. Primers were developed as described by Bagdonaitė et al. (2024). Sequences used for primer design can be found in Table S2. In brief, multiple sequences representing a range of targeted and non‐targeted species were downloaded from GenBank and our own data and aligned in the Geneious Pro R10 software (https://www.geneious.com) using the multiple alignment function, after which consensus sequences for each species were generated. Then, species alignment sequences were used to identify fragments with low variation and therefore suitable for primer development. The Geneious primer design option was used to generate candidate primer pairs. Primer pairs were evaluated through visual alignment with the consensus sequences and primer parameters to ensure optimal efficiency of the PCR reactions. Following this, both in vitro and in silico primer validation steps were carried out on all four genes. In vitro validation included visual assessment of the primers using Integrated DNA Technologies (IDT) PrimerQuest tool (PrimerQuest program, IDT, Coralville, Iowa, USA), and primer‐blast on the NCBI website (https://www.ncbi.nlm.nih.gov/tools/primer‐blast/). Then, in silico validation was carried out by performing multiple qPCRs on DNA extracted from corresponding species, ensuring that all genes were amplified at the same temperature and that each primer set only amplified a single product. This resulted in two primer sets: gene set 1 targeting 16S (mtDNA) and GAPDH (nDNA) fragments across all passerines used in this study; gene set 2 targeting 12S (mtDNA) and ELF1 (nDNA) fragments across the finch (family Fringillidae) samples.

**TABLE 2:** Primers used in this study.

Gene	Sequence	Fragment length (bp)	Annealing temperature
16S (mtDNA)	Forward 5′‐ATTATTGAGCGAACCCGTCTC‐3′ Reverse 5′‐TTCACAGGCAACCAGCTATC‐3′	98	55°C
GAPDH exon 9 (nDNA)	Forward 5′‐CATCACAGCCACACAGAAGA‐3′ Reverse 5′‐CTCCAGTAGATGCTGGGATAATG‐3′	98	
12S (mtDNA)	Forward 5′‐CACTATGCCTGGCCCTAAAT‐3′ Reverse 5′‐CCGCCAAGTCCTTAGAGTTT‐3′	96	
ELF 1 exon 8 (nDNA)	Forward 5′‐CAGAAACCAACCAAGCAGAAC‐3′ Reverse 5′‐TTAGCAGGCTTCGAGTTTCC‐3′	96	

*Note:* Universal across all study species gene set 1 (16S/GAPDH), and finch‐specific gene set 2 (12S/ELF 1). All amplified fragments were 96–98 bp long and had the same annealing temperature of 55°C.

### 2.4 ddPCR

ddPCR reactions were performed on a Bio‐Rad QX200 ddPCR System, and all samples were quantified in triplicate. 16S and GAPDH were always quantified on the same plate, as were 12S and ELF1, to ensure that the mtDNA to nDNA ratio was calculated, avoiding any effects that could be introduced when setting up reactions on different plates (Pinheiro et al. 2012). Each reaction was conducted in a final volume of 20 μL as follows: 10 μL Bio‐Rad ddPCR EvaGreen supermix, 0.25 μL of forward and reverse primers (10 μM each), 4 μL of template DNA for mtDNA quantification and 6 μL of template DNA for nDNA quantification, 5.5 μL of ddH20 for reaction targeting mtDNA, and 3.5 μL of ddH20 for reaction targeting nDNA. A larger volume of DNA sample was used for nDNA quantification to ensure efficient detection and quantification, as our previous tests have shown that spermatozoa have a very low nDNA copy number. After thorough vortexing, each reaction was pipetted into a DG8 Droplet Generator Cartridge and mixed with 70 μL of Droplet Generation Oil for EvaGreen. A QX200 Droplet Generator (Bio‐Rad) was used to generate up to 20,000 droplets per well, after which 40 μL of the emulsion was carefully pipetted to a ddPCR 96‐well plate, covered with a pierceable foil, and sealed with a PX1 PCR Plate Sealer (Bio‐Rad). We performed the PCR reactions on a Bio‐Rad CFX96 Real‐Time System (Bio‐Rad Laboratories, California, United States). The conditions were as follows: 10 min at 95°C, 40 cycles of 30s denaturation at 94°C and 1 min annealing at 55°C, with a ramp‐up rate of 2°C/s, followed by 10 min at 98°C and a hold at 8°C. A QX200 droplet reader (Bio‐Rad) with Bio‐Rad QuantaSoft software (v.1.7.4.0917) was used to read the droplets and obtain quantification information.

First, we quantified mtDNA copy number of the seven finch species using a varying number of individuals per species (*N* = 2–4) (Table 3) and the finch‐specific primers (12S/ELF1). mtDNA copy number in these samples was also quantified using the universal primer set (16S/GAPDH). Quantification values obtained for both gene sets were used to assess the comparability of mtDNA copy number when different genes are quantified and as a control step to identify potential primer biases or other specific ddPCR run effects.

**TABLE 3:** Summary of the quantification results across species.

Species	Set 1 (16S/GAPDH)	Set 2 (12S/ELF1)	Number of individuals: Set 1/Set 2
Yellowhammer	28		4/—
Common reed bunting	60		4/—
Eurasian siskin	27 (14)	13	10/4
Common redpoll	15	15	4/4
European greenfinch	22	21	4/4
Hawfinch	30	27	4/4
Eurasian chaffinch	36 (34)	30	4/2
Brambling	37 (39)	34	4/3
Eurasian bullfinch	50 (42)	45	4/3
Meadow pipit	19		3/—
Bluethroat	18		4/—
Coal tit	15		4/—
Eurasian blue tit	20		4/—
Great tit	22		4/—
House sparrow	9		4/—
Willow warbler	56		4/—
Goldcrest	38		4/—
Eurasian wren	26		4/—
Fieldfare	17		2/—

*Note:* The ratio between mitochondrial 16S and nuclear GAPDH (gene set 1) was quantified for all 19 species. The same samples of the family Fringillidae were also quantified with 12S/ELF1 (gene set 2). If the number of individuals per species used with gene set 1 and gene set 2 was different, the average mtDNA ratio of only the samples quantified with both gene sets is indicated in parentheses for direct comparison. The number of individuals varied across species and is indicated in the final column.

Then, we quantified 16S/GAPDH in additional finch individuals to increase the sample number to four (except for siskin, where *N* = 10) as well as a number of species from other families (Table 3). Four individuals per species were quantified when available (with the exceptions of meadow pipit and fieldfare). We did not sample extensively within species as the main goal of this study was to examine the relationship between mtDNA copy number and midpiece length across and not within species.

Across all plates and markers, fluorescence thresholds for positive signals were set at 10,000, and all droplets above this value were counted as positive events. Each plate included one to three negative controls per gene, ensuring that there was no contamination. The ratio of mtDNA to nDNA was generated by first calculating the individual average for each gene (from three technical replicates); then, we calculated a species average, and the final ratio was calculated as 1.5 × mtDNA/nDNA to account for the different DNA input volumes for the reactions. We will refer to this ratio as mtDNA copy number further in the text.

### 2.5 Phylogeny

We generated two phylogenetic trees: one for all 19 species used in the present study and one without the bullfinch based on the time‐calibrated molecular phylogeny of all extant avian taxa (Jetz et al. 2012), following the method described by Rowe et al. (2015). Newick files are available in the Dryad Digital Repository. We downloaded 1000 randomly selected phylogenetic trees from www.birdtree.org. We then used TreeAnnotator version 2.6.3 (BEAST; Drummond et al. 2012) to summarize the trees onto a single maximum clade credibility (MCC) tree with mean node heights.

### 2.6 Statistical Analyses

All statistical analyses were performed in Rstudio (Posit Team 2024), using R Statistical software (v4.3.2; R Core Team 2023). All raw data are available in the Dryad Digital Repository. Data import, curation, and visualization were done using tidyverse v2.0.0 (Wickham et al. 2019) and ggrepel v0.9.4 (Slowikowski 2024) packages. All plots were generated using ggplot2 v3.4.4 (Wickham et al. 2024).

To test for the repeatability of ddPCR quantification among the technical replicates (*N* = 3 per individual) and mtDNA copy number estimation within species (*N* = 2–10 individuals per species), we used the bootstrapping approach for Gaussian data implemented in the rptR package, v. 0.9.22 (Stoffel et al. 2017) with 1000 permutations. A Pearson correlation test was carried out to compare the quantification results of 16S/GAPDH versus 12S/ELF1 obtained for the seven finch species. In addition, 16S and GAPDH concentrations (copies/μL) were ln‐transformed, and Levene's test for homogeneity of variance was performed to test if both 16S and GAPDH had equal variances and then the variation across species was plotted.

To investigate the relationship between mtDNA copy number and midpiece length across species, we used a generalized least‐squares approach in a phylogenetic framework (PGLS), using the following R packages: ape v5.8 (Paradis and Schliep 2019), nlme v3.1‐163 (Pinheiro et al. 2023), and phytools v2.1‐1 (Revell 2012). We compared polynomial and linear regression models both on a complete dataset and excluding the bullfinch. In these regressions, we used species averages of ln‐transformed mtDNA copy number per spermatozoon (16S normalized against GAPDH) as the dependent variable, and ln‐transformed midpiece length and its square as predictors. We estimated the phylogenetic signal by calculating Pagel's *λ* (Pagel 1999); 0 indicates no phylogenetic signal, while 1 shows a strong association between species. If the model estimated *λ* > 1, we ran the model again, fixing *λ* at 1. Models were compared using the Akaike Information Criteria (AIC).

To investigate the relationship between mtDNA copy number and midpiece length within species, we used a subset of our data (48 individuals) for which we had midpiece length measurements for each individual. We *z*‐transformed our data—calculated the mean ln‐transformed midpiece length for each species and the deviation for each individual. We then used a linear mixed‐effects (lme) model from the R package nlme v3.1‐163 (Pinheiro et al. 2023), in which ln‐transformed individual mtDNA copy number was treated as the dependent variable and the midpiece length deviation was treated as a predictor, with species as a random effect.

## 3 Results

### 3.1 Quantification

**FIGURE 1:**
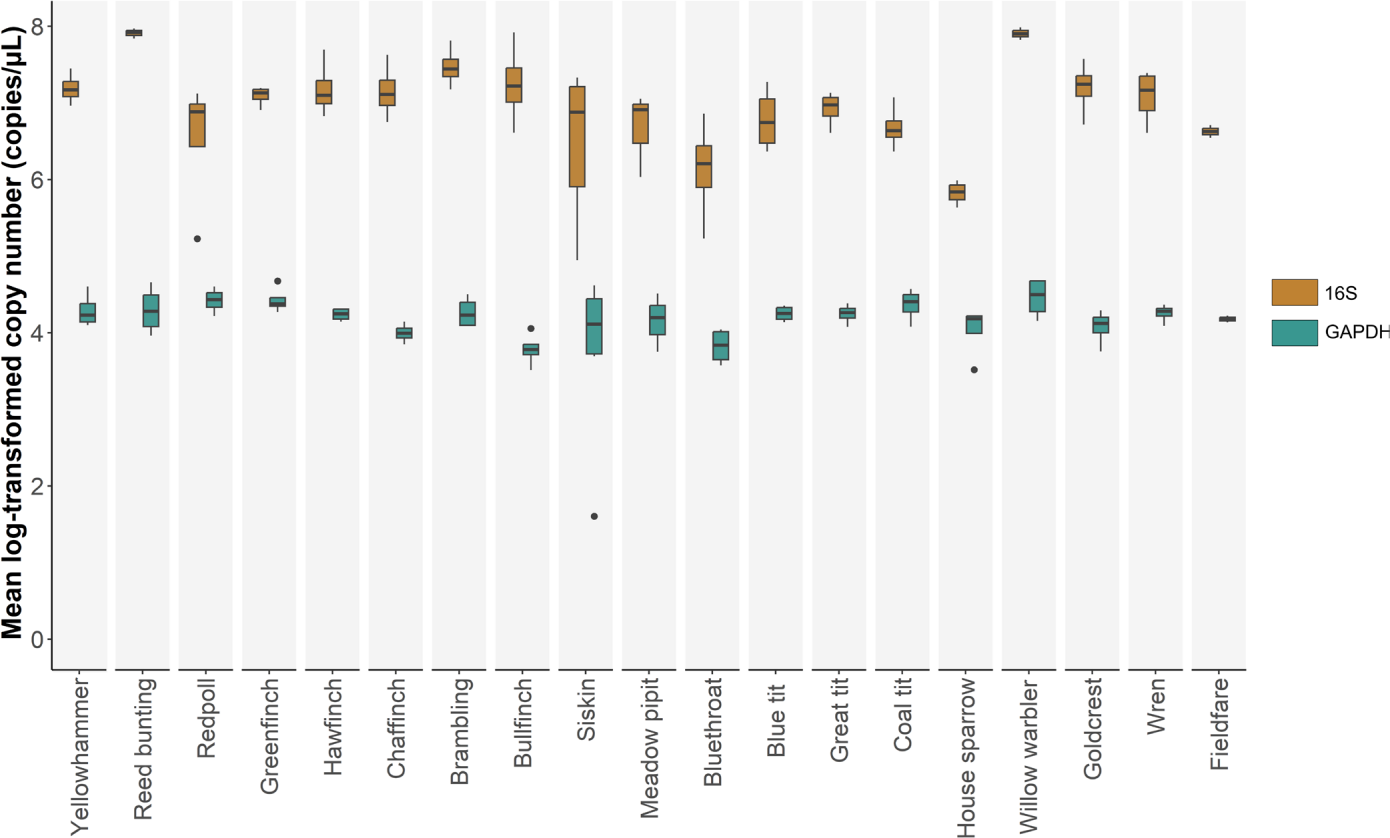
Comparison of log‐transformed mitochondrial 16S (orange) and nuclear GAPDH (blue) quantification across species.

A total of 674 ddPCR reactions were performed across 10 96‐well ddPCR plates, including 56 negative controls that showed no amplification. There was a very high repeatability of the ddPCR‐based DNA quantification of all four genes used in the study, with repeatability estimates *R* > 0.98 across technical replicates of each sample (Table 4). We found that ejaculates from all species contained a significantly higher copy number of mitochondrial DNA than nuclear DNA (paired *t*‐test, *t* = 13.63, *p* < 0.005; Figure 1).

**TABLE 4:** Repeatability of ddPCR‐based DNA quantification across technical replicates and species.

	Marker	Repeatability (*R*) ± SE
Repeatability across technical replicates	16S (copies/μL)	0.998 ± 0.000432
	GAPDH (copies/μL)	0.966 ± 0.0067
	12S (copies/μL)	0.997 ± 0.00108
	ELF1 (copies/μL)	0.985 ± 0.00626
Repeatability within species	16S/GAPDH (copies/spermatozoon)	0.579 ± 0.113
	12S/ELF1 (copies/spermatozoon)	0.575 ± 0.212

*Note:* The table provides repeatability estimates of DNA quantification; (1) within individuals (three technical replicates) for four target genes (16S, GAPDH, 12S, and ELF1); (2) within species (2–10 individuals) for individual mtDNA copy number per spermatozoon (16S/GAPDH and 12S/ELF1).

**FIGURE 2:**
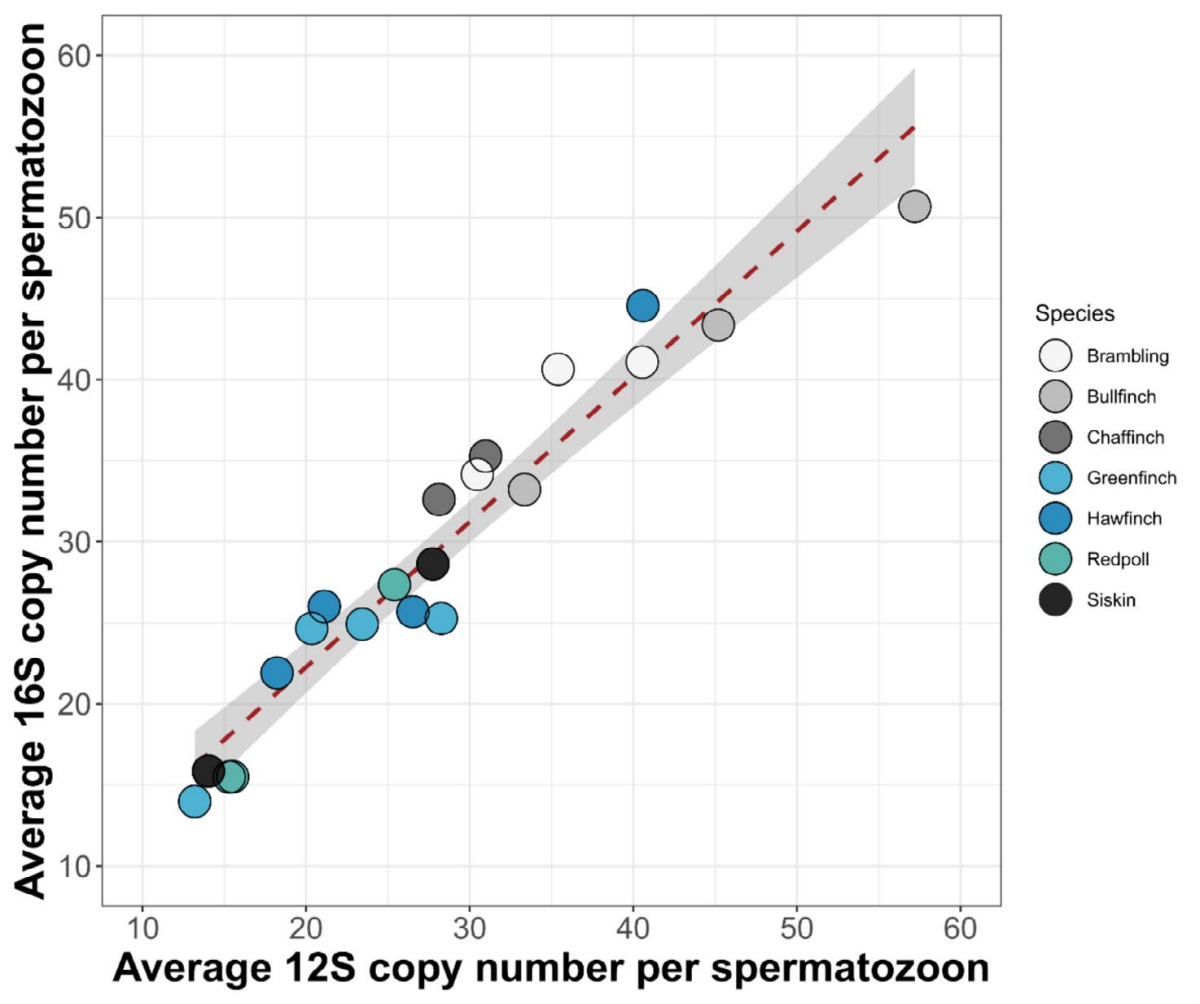
Comparison of mtDNA copy number quantified using two different primer sets (16S/GAPDH and 12S/ELF1) for the finch family Fringillidae. Each point represents the mean copy number per sample (calculated from three technical replicates). The figure shows that quantification results obtained for both mitochondrial genes are highly similar (Pearson correlation coefficient = 0.976).

The average copy number per spermatozoon (mitochondrial 16S normalized against GAPDH) was variable across the 19 species and ranged from 9 in house sparrow to 60 in reed bunting (Table 4). Quantification of 12S (normalized against ELF1) in the finch (family Fringillidae) samples showed similar variation (Table 4). We compared 16S and 12S copy numbers in finches and found them to be highly similar (Pearson correlation coefficient = 0.976, *p* < 0.005; Figure 2). This result suggests that there was no bias between primers used in this study. However, within species, both 16S (normalized against GAPDH) and 12S (normalized against ELF1) copy numbers were highly variable (Table 4).

Variation in mitochondrial 16S was significantly greater than the variation in nuclear GAPDH across the 19 species in our dataset (Levene's test, *F* = 15.5, *p* < 0.005; Figure 1). The low variability of nuclear GAPDH across species should reflect the similar loading amount of DNA since nuclear DNA contributes more to the total concentration. Therefore, the variation we see in 16S copy number is a result of the varied mtDNA concentration in spermatozoa. Additionally, a one‐way ANOVA revealed that there is significantly more variation in mtDNA copy number among species than within, despite a high within‐species variability (*F* = 6.40, *p* < 0.001).

### 3.2 The Relationship Between mtDNA Copy Number and Midpiece Length

**FIGURE 3:**
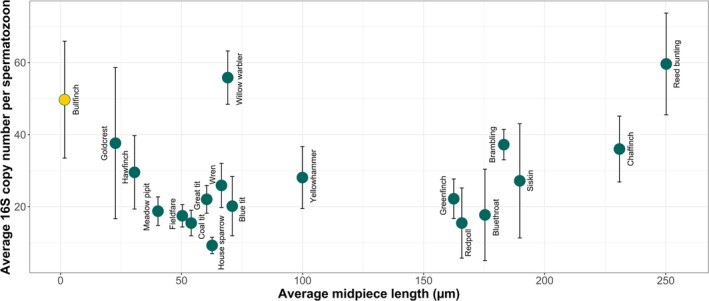
Relationship between mtDNA copy number per spermatozoon (expressed as ratio between mitochondrial 16S and nuclear GAPDH) and average midpiece length across passerines. For each species, we quantified DNA of at least two individuals. The bars indicate standard deviation within species. We used the ln‐transformed measurements in our statistical analyses; however, here we plot untransformed values. Bullfinch is in yellow as this species is known to have very different sperm morphology than the rest of our study species.

The relationship between mean mtDNA copy number and sperm midpiece is visualized in Figure 3. The statistical tests, on ln‐transformed values, are given in Table 5. Neither the polynomial regression nor the linear regression gave any support for a positive association between midpiece length and mtDNA copy number among the 19 species. In fact, the polynomial regression showed a marginally significant negative association for the linear term. No significant relationships were shown when we excluded the bullfinch, which has a deviant structure and an extremely short midpiece (Table 5).

**TABLE 5:** Results of pgls models.

	Model	Predictor	Value	SE	*t*	*p*	AIC	Pagel's Λ
All species	1	Intercept	4.58	0.60	7.65	0.00	**30.11**	1
		Midpiece length^2^	0.10	0.05	1.90	0.08		
		Midpiece length	−0.76	0.34	−2.20	0.04		
	2	Intercept	3.76	0.44	8.51	0.00	31.96	1
		Midpiece length	−0.13	0.09	−1.37	0.19		
No bullfinch	3	Intercept	11.51	4.39	2.62	0.02	**27.33**	1
		Midpiece length^2^	0.45	0.22	2.00	0.06		
		Midpiece length	−3.90	2.00	−1.95	0.07		
	4	Intercept	2.83	0.77	3.69	0.00	29.60	1
		Midpiece length	0.09	0.18	0.53	0.60		

*Note:* Models 1–4 investigate the association between ln‐transformed mtDNA copy number and ln‐transformed midpiece length across species. Models 1–2 use all 19 species, and models 3–4 do not include the bullfinch. In all four models, Pagel's Λ was fixed at 1 due to previous runs estimating Λ > 1. Both with and without the bullfinch, the better fitting model was the polynomial (lower AIC value) (models 1 and 3). AIC values in bold indicate the better fitting models for the two sets of data.

Among the 14 species where we had individual midpiece length measurements, there was no indication that a longer midpiece was associated with a higher mtDNA copy number within species (SE = 1.68, df = 33.00, *F* = 1.19, *p* = 0.24).

## 4 Discussion

We have shown that there is consistent variation in sperm mtDNA copy number among 19 species of songbirds, but this variation is not positively associated with variation in the sperm midpiece length. Our ddPCR method for mtDNA copy number estimation appears robust, with high technical repeatability and consistent results between two different sets of primers used. Midpiece length is also a good proxy for midpiece volume in songbirds, as shown in a previous study (Cramer et al. 2022). We are therefore confident in concluding that songbirds with a longer midpiece do not have more copies of mtDNA in their spermatozoa.

Our main results contrast with the previously shown relationship between the midpiece length and mtDNA copy number in the zebra finch (Knief et al. 2021). While it has been shown that the difference in copy number between sperm with short and long midpieces in this species is roughly 5 per spermatozoon, the midpiece length differs only by 7 μm. In contrast, the species examined in our study exhibit a substantial difference in midpiece length representing a nearly 100‐fold variation, but the variation in copy number among these species was relatively modest, differing only three to five times. The lowest average mtDNA copy number was observed in house sparrows—9 copies per spermatozoon, while the highest—60 mtDNA copies per spermatozoon, was recorded in the species with the longest midpiece—reed bunting. Surprisingly, the species with the shortest midpiece—the bullfinch had an average of 50 mtDNA copies per spermatozoon. However, the sperm morphology of *Pyrrhula* bullfinches differs from other passerines in major respects (Birkhead et al. 2006; Lifjeld et al. 2013). In the Eurasian bullfinch, spermatozoa exhibit putative neoteny, resembling immature spermatozoa or spermatids. TEM images show that the midpiece in this species is formed by a small group of mitochondria (in contrast to one long mitochondrion typically observed in passerines), which strongly suggests that the final stages of spermatogenesis are suppressed in these species; however, this remains an untested hypothesis (Birkhead et al. 2007). The high number of mtDNA in the bullfinch sperm observed in the present study, combined with the findings of (Birkhead et al. 2007), could indicate that mtDNA reduction in passerines happens at the later stages of spermiogenesis, and if these stages are skipped in the species, mtDNA copy number could remain especially high relative to the midpiece length.

A very high mtDNA copy number was also observed in the willow warbler, making it stand out as an outlier among other species (Figure 3). Unlike in the bullfinch, no unusual sperm morphometrics have been reported for this species, and the spermatozoa generally follow the morphology observed in other passerines (Støstad et al. 2016), it is difficult to speculate what causes such a high mtDNA copy number in this species. The willow warbler is the only representative of the family Phylloscopidae in our dataset and is rather distantly related to the other families, which could explain why it has such a high mtDNA copy number compared to other species with similar midpiece lengths. Therefore, further studies including more representatives of the Phylloscopidae and an increased sample size per species will be necessary to better understand such results.

As shown in some non‐avian vertebrates and invertebrates, mitochondrial DNA content undergoes a strong reduction at the final stages of spermatogenesis as a pre‐fertilization barrier of paternal mtDNA transmission (DeLuca and O'Farrell 2012; Rantanen and Larsson 2000; Wolff and Gemmell 2008). Even though mitochondrial functionality and an intact mitochondrial membrane are essential for all normal cell functions, and especially energetic needs, sperm mtDNA has been shown to be susceptible to damage by reactive oxygen species (ROS) and thus, the mtDNA reduction is seen as evolutionarily advantageous (Faja et al. 2019; Luo et al. 2013). Studies on mammals have documented a negative association between mtDNA abundance and sperm quality, or swimming speed (Boguenet et al. 2022; Luo et al. 2013). In contrast, studies on zebra finches have shown a positive association between mtDNA copy number and midpiece length (Knief et al. 2021), and a likely positive association between midpiece length, swimming speed, and motility in this species (Knief et al. 2017). One potential explanation for such discrepancy between mammals and passerine birds could be the fact that dominant energy sources are different in these groups (glycolysis in mammals, OXPHOS in birds) (Rowe et al. 2013) and thus the removal of mtDNA in birds is less crucial; however, further proof is still lacking and more comparative studies in passerine birds are needed.

Comparative studies in mammals have shown that the most motile spermatozoa eliminate their mtDNA before fertilization (Boguenet et al. 2022; Guo et al. 2017; Luo et al. 2013). Despite the fact that most motile spermatozoa have less mtDNA, they show a higher mitochondrial activity as well as higher contents of mtDNA‐encoded and nDNA‐encoded proteins essential for OXPHOS, as well as more pronounced mitochondrial membranes (cristae) (Guo et al. 2017), demonstrating the disconnect between mtDNA copy number and the functioning of the mitochondria in the sperm midpiece. While midpiece length is positively correlated with mitochondrial volume in passerines (Cramer et al. 2022) and longer midpieces are predicted to have been formed by more fused mitochondria (Cardullo and Baltz 1991), an intraspecific study on zebra finches shows that sperm with shorter midpieces had a higher concentration of ATP (Bennison et al. 2016). This finding contrasts with the results published by (Rowe et al. 2013), which show that across 23 passerine species spermatozoa with longer midpieces have more intracellular ATP. However, our findings together with the studies mentioned above suggest that mtDNA copy number cannot be used as a proxy for midpiece length in passerines.

As in the present study, studies of mtDNA content in other animal groups have shown a substantial variation in mtDNA copy number between individuals of the same species. When mouse ( *Mus musculus* ) sperm cells were categorized by their motility, the most motile spermatozoa contained as little as 1.29 copies of mtDNA per cell, whereas the less motile cells had up to 45.93 copies of mtDNA per sperm (Luo et al. 2013). A very similar pattern has been observed in a human study (Boguenet et al. 2022). Differences in mtDNA copy number between spermatozoa with normal sperm parameters and in patients with severe oligoasthenospermia (a condition when the total number and motility of spermatozoa is low), one of the main causes of low fertility in humans (Zhang et al. 2020), were analyzed, confirming that mtDNA copy number was significantly higher in abnormal sperm cells. A comparative study of 14 chinook salmon (*Oncorhynchus tshawytscha*) individuals reported a highly variable mtDNA copy number, ranging from 2.77 copies to 9.42 copies per spermatozoon (Wolff and Gemmell 2008). Furthermore, pre‐fertilization elimination of mtDNA from spermatozoa has also been shown in the fruit fly (DeLuca and O'Farrell 2012) which can reduce the mtDNA content to less than one copy per cell according to the study. As in the vertebrate studies mentioned above, fruit flies were shown to retain the mitochondrial membranes necessary for normal cell function and energy production (McBride et al. 2006) despite removing mtDNA. Active digestion and reduction of mtDNA have also been shown during spermatogenesis of the Japanese medaka fish, where a fivefold reduction of mtDNA copy number in mature spermatozoa was reported (Nishimura et al. 2006).

While the exact mechanisms regulating mtDNA copy number in wild passerines remain unclear and more comparative studies are necessary to disentangle them, our study provides important insights. In accordance with mammal and invertebrate data, we show that there is substantial individual variation in mtDNA copy number within songbird species. Furthermore, our results demonstrate that the overall differences in mtDNA copy number across species are not representative of their highly diversified midpiece lengths. Contrary to our expectation that we would find a positive correlation between mtDNA copy number and midpiece length, it appears that the mtDNA content of songbird spermatozoa is reduced during spermatogenesis, potentially within a conserved range across taxa.

In conclusion, our results demonstrate for the first time that mitochondrial DNA abundance in mature spermatozoa does not correlate with the midpiece length in Passerides songbirds. If the hypothesis that longer midpieces contain more fused mitochondria is correct (Cardullo and Baltz 1991), our findings suggest that mitochondrial DNA is removed at the late stages of spermatogenesis so that the copy number retained in mature spermatozoa is small with low variation among songbird species. However, more comparative studies with increased sample sizes as well as comparisons of mtDNA copy number in mature versus immature spermatozoa are needed to further our understanding of this issue.

## Author Contributions

**Erica H. Leder:** conceptualization (equal), data curation (equal), writing – review and editing (equal). **Jan T. Lifjeld:** conceptualization (equal), data curation (equal), writing – original draft (equal). **Quentin Mauvisseau:** conceptualization (equal), data curation (equal), formal analysis (supporting), writing – review and editing (equal). **Arild Johnsen:** conceptualization (equal), data curation (equal), writing – review and editing (equal). **Laima Bagdonaitė:** conceptualization (equal), data curation (equal), formal analysis (lead), writing – original draft (lead), writing – review and editing (equal).

## Conflicts of Interest

The authors declare no conflicts of interest.

## Supporting information


Table S1.
Table S2.

## Data Availability

All raw data, Supporting Information, R code, and phylogenetic trees can be accessed on Dryad Repository https://doi.org/10.5061/dryad.0rxwdbsbb.
